# Use of Artificial Intelligence in the Prediction of Chiari Malformation Type 1 Recurrence After Posterior Fossa Decompressive Surgery

**DOI:** 10.7759/cureus.60879

**Published:** 2024-05-22

**Authors:** Vincent King, Sidong Liu, Carlo Russo, Mudith Jayasekara, Marcus Stoodley, Antonio Di Ieva

**Affiliations:** 1 Neurosurgery, Liverpool Hospital, Sydney, AUS; 2 Medicine, Health, and Human Sciences, Computational NeuroSurgery (CNS) Lab, Macquarie Medical School, Macquarie University, Sydney, AUS; 3 Center of Health Informatics, Macquarie University, Sydney, AUS; 4 Engineering Science, Institute of Biomedical Engineering, University of Oxford, Oxford, GBR; 5 Medicine, Health, and Human Sciences, Macquarie Medical School, Macquarie University, Sydney, AUS; 6 Neurosurgery, Nepean Blue Mountains Local Health District, Sydney, AUS; 7 Center for Applied Artificial Intelligence, School of Computing, Macquarie University, Sydney, AUS

**Keywords:** machine learning, posterior fossa surgery, recurrence, deep learning, chiari malformation

## Abstract

Purpose

The purpose of this study was to train a deep learning-based method for the prediction of postoperative recurrence of symptoms in Chiari malformation type 1 (CM1) patients undergoing surgery. Studies suggest that certain radiological and clinical features do exist in patients with treatment failure, though these are inconsistent and poorly defined.

Methodology

This study was a retrospective cohort study of patients who underwent primary surgical intervention for CM1 from January 2010 to May 2020. Only patients who completed pre- and postoperative 12-item short form (SF-12) surveys were included and these were used to classify the recurrence or persistence of symptoms. Forty patients had an improvement in overall symptoms while 17 had recurrence or persistence. After magnetic resonance imaging (MRI) data augmentation, a ResNet50, pre-trained on the ImageNet dataset, was used for feature extraction, and then clustering-constrained attention multiple instance learning (CLAM), a weakly supervised multi-instance learning framework, was trained for prediction of recurrence. Five-fold cross-validation was used for the development of MRI only, clinical features only, and a combined machine learning model.

Results

This study included 57 patients who underwent CM1 decompression. The recurrence rate was 30%. The combined model incorporating MRI, pre-operative SF-12 physical component scale (PCS), and extent of cerebellar ectopia performed best with an area under the curve (AUC) of 0.71 and an F1 score of 0.74.

Conclusion

This is the first study to our knowledge to explore the prediction of postoperative recurrence of symptoms in CM1 patients using machine learning methods and represents the first step toward developing a clinically useful prognostication machine learning model. Further studies utilizing a similar deep learning approach with a larger sample size are needed to improve the performance.

## Introduction

Chiari malformation type 1 (CM1) is a disorder that is characterized by cerebellar tonsillar herniation below the foramen magnum. The mainstay of treatment for symptomatic patients with CM1 is surgical decompression of the posterior fossa. Around 75-90% of CM1 patients experience improvement of physical signs and symptoms following posterior fossa decompression but up to a third of patients experience recurrence, defined as persistence or return of symptoms after surgery, with 4-13% of patients requiring reoperation [1-3]. Diagnosis of CM1 recurrence currently relies on clinical testing and neuroimaging (primarily, magnetic resonance imaging, MRI). Various studies have attempted to identify the factors that increase the risk of symptom recurrence after posterior fossa decompression. Current literature has highlighted clinical and radiological features associated with recurrence in CM1 and these have been incorporated into prediction systems including the Chiari Severity Index and points-based algorithm of Thakar et al. [4,5]. Feghali et al., upon external validation of these systems, concluded that prediction of treatment failure in CM1 remains difficult and inconsistent [6]. Attempts have been made with artificial intelligence (AI), including machine learning and deep learning methodologies, to aid in the diagnosis of CM1 and understand the diagnostic factors, with the aim of identifying potential features that may predict its symptomatic recurrence [7].

AI will continue to expand and has the potential to be an important tool in clinical neurosurgery, though it is in its infancy. In this study, we applied machine learning and deep learning techniques on MRI data together with clinical features to predict recurrence in CM1 patients who have undergone posterior fossa decompression as a novel way to assist in patient selection, clinical decision-making, and outcome prediction. To our knowledge, there has not been a study using AI for the prediction of CM1 recurrence after surgery.

## Materials and methods

Study participants

This retrospective cohort study included patients who had a diagnosis of CM1 from January 2010 to May 2020 with appropriate electronically accessible preoperative imaging and who underwent primary surgical intervention at our institution, a single quaternary neurosurgical center in Sydney, Australia. This dataset was from a single experienced neurosurgeon at a single institution. This study was approved by the University Human Research Ethics Committee. 

Only patients who had completed a 12-item short-form survey (SF-12) preoperatively and postoperatively were included in the study. Patient characteristics and clinical information were collected, including gender, age at surgery, headache, limb pain, numbness, limb paraesthesia, limb weakness, vertigo, nystagmus, duration of symptoms, and radiological characteristics, such as cerebellar ectopia and presence or absence of syringomyelia. Patients had been diagnosed and treated by practicing radiologists (with expertise in neuroradiology) and neurosurgeons and as such there were no patients included with possible alternative diagnoses such as spontaneous intracranial hypotension. 

Neuroimaging and MRI pre-processing

Images were acquired on a 3 Tesla MR unit (Siemens Magnetom Verio, Erlangen, Germany) with the following parameters: TR/TE/TI: 2000/8.4/800 ms, flip angle: 150°, field of view: 240×240 mm^2^, slice thickness: 4.5 mm, and matrix: 320×320. T1 fluid-attenuated inversion recovery (FLAIR) sequence in the sagittal plane and cine phase-contrast MRI were used for the study. All imaging data were saved to secure private servers for the computational analysis. All MRI volumes were anonymized and underwent Digital Imaging and Communications in Medicine (DICOM) to Neuroimaging Informatics Technology Initiative (NIfTI) conversion and intensity normalization. 

Surgical procedure

Posterior fossa decompression and expansile duroplasty were performed on all patients under general anesthesia. A midline incision was made extending from the occipital protuberance to the C2 spinous process, which exposed the edge of the occipital bone and C1 posterior arch. Then, the inferior part of the occipital bone and the posterior lamina of C1 were removed to achieve a bony decompression. Durotomy was performed in a Y shape. Expansile duraplasty was performed by using occipital pericranium, sutured for dural grafting. 

Follow-up

Patients were followed up after four weeks and up to >18 months. The clinical outcomes were evaluated based on their SF-12 physical component scale (PCS) results completed at each follow-up and compared with their preoperative result. Recurrence was defined as a worsening of the SF-12 PCS compared to their preoperative score at the most recent follow-up, which was at least more than four weeks after surgery.

Data augmentation

**Figure 1:**
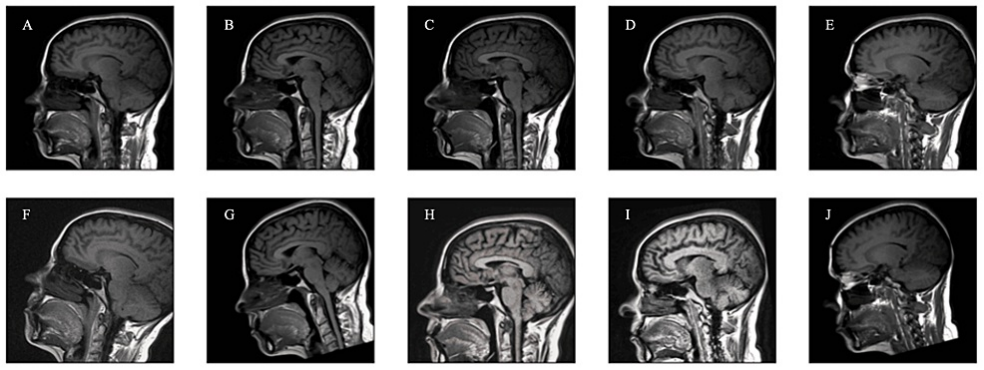
Examples of images following data augmentation in the dataset A) Original MRI slice. (B-J) Examples of multiple augmented versions that were created for each MRI slice. The augmentation included a range of techniques such as random shifts of 10%; scaling by 10%; rotation of ±20°; cropping, sharpening, and blurring; perspective transformations; addition of Gaussian noise; adjustments in brightness and hue saturation value levels; and gamma transformations. MRI, magnetic resonance imaging

To improve the training process with a relatively small amount of data, the training and validation datasets were augmented using the Albumentations library (version 1.3.1) [8]. The test set was not augmented. The augmentation included a range of techniques such as random shifts of 10%; scaling by 10%; rotation of ±20°; cropping, sharpening, and blurring; perspective transformations; addition of Gaussian noise; adjustments in brightness and hue saturation value levels; and gamma transformations. Some examples are shown in Figure 1.

Data augmentation was performed at the image level such that multiple augmented versions were created for each MRI slice. Data augmentation was implemented for both the minority class (i.e., the "recurrence" patients) and the majority class (i.e., the "improved" patients). To address the dataset’s imbalanced distribution, the minority class underwent a 10-fold augmentation, whereas the majority class underwent a five-fold augmentation.

Deep learning-based recurrence prediction 

Using these volumes, an MRI-only deep learning-based model was developed to predict CM1 symptomatic recurrence after posterior fossa decompression. The sagittal FLAIR volumes were transformed into stacks of images, with each image having a resolution of 512x512 pixels. A ResNet50 model, pre-trained on the ImageNet dataset, was then used to extract a 1024-dimensional feature vector from each image. Therefore, each FLAIR volume could be represented by a sequence of feature vectors. The ResNet50 pre-trained model is one of the most used convolutional neural network models used in the biomedical sciences for transfer learning. It was not re-trained or fine-tuned. The use of the model allowed for efficient extraction of the low-level features in the images.

Clustering-constrained attention multiple instance learning (CLAM), a weakly supervised multi-instance learning framework, was chosen as the deep learning-based pipeline for classification [9]. CLAM is traditionally used for histology analysis and provides whole slide image classification using slide-level labels without any region of interest extraction or patch-level annotations. It is effective for our dataset as each MRI scan only carries a volume-level label, either "improved" or "recurrence," and lacks annotations at the section or pixel level. 

The model uses attention-based learning to identify subregions of high diagnostic value to classify the whole volume. The model was trained using the following hyperparameters: the number of epochs was set to 200, with early stopping implemented after 20 epochs. The Adam optimizer was used, with an initial learning rate of 1x10-4 and weight decay of 1x10-5. The model type was set to multiple branches. Batch size was set to eight, and weighted sampling was enabled to mitigate the effects of an imbalanced class distribution. For loss functions, cross-entropy was used for bag loss, while support vector machine (SVM) was employed for instance loss.

Clinical feature-based recurrence prediction

In addition to this MRI-only model, four clinical feature-based models were developed using a combination of age, gender, preoperative SF-12 PCS, extent of cerebellar ectopia, presence or absence of a syrinx, cine MRI score (either A, B, C, or D), and preoperative symptoms. A summary of the definitions of the different cine scores is seen in Table 1. Symptoms included the presence of headaches, limb pain, limb paraesthesia, limb weakness, numbness, vertigo, and nystagmus. For these models, the logistic regression algorithm was used to train the classifiers, using the limited-memory BFGS algorithm as the optimizer and the max number of iterations set to 1000 [10-12].

**Table 1:** Summary of different cine MRI scores MRI, magnetic resonance imaging

Cine score	Definition
A	Normal hindbrain CSF flow
B	Abnormal CSF flow dorsal to the cervicomedullary junction
C	Abnormal CSF flow both ventral and dorsal to cervicomedullary junction
D	Abnormal CSF flow ventral to cervicomedullary junction

Logistic regression classification models were further used for a combined MRI and clinical feature-based model.

Performance evaluation 

K-fold cross-validation was used to estimate the precision of the model’s performance, where k was five in this study to randomly partition the initial dataset. Given the small sample size of our dataset, if k-fold cross-validation was not used, the held-out test dataset would be prone to random bias. Within each of the five folds, the dataset was split randomly into training, validation, and test datasets in a ratio of 60:20:20, respectively. Stratified random sampling was utilized so the ratio improved and recurrence between folds was stable. In each iteration of the five-fold cross-validation for the MRI-only model, a neural network following the CLAM framework was trained. This process necessitates the use of a validation set to ascertain the completion of training, specifically, identifying when the network achieves peak performance on the validation set. Following this phase, the fully trained model underwent evaluation on the held-out test set that did not have any data augmentation. The mean AUC and standard deviation (SD) of the five runs were calculated for each model. 

## Results

In total, 142 patients underwent CM1 decompression surgery during the study period. After excluding patients without both preoperative and postoperative SF-12 PCS, 57 patients remained. 

Table 2 shows the characteristics of the 57 participants included in this study. At their follow-up, 40 (70%) patients had improvement in symptoms while 17 (30%) had a recurrence of their symptoms based on the SF-12 PCS. There was a high proportion of females in both the "improved" and "recurrence" groups, 36 (90.0%) and 15 (88.2%) patients, respectively. The mean preoperative SF-12 PCS was 28.7 (interquartile range: 22.4-34.3) for the "improved" group and 35.6 (interquartile range: 30.3-36.4) for the "recurrence" group. The mean cerebellar ectopia was 11.0 mm (interquartile range: 7.8-14.2) for the "improved" group and 8.2 mm (interquartile range: 6.0-10.8) for the "recurrence" group. Limb pain was experienced as a symptom preoperatively by 11 (27.5%) patients in the "improved" group and one (5.9%) patient in the "recurrence" group. 

**Table 2:** Patient characteristics, preoperative scores, and presenting symptoms stratified by whether the patients improved or had a recurrence of symptoms MRI, magnetic resonance imaging; PCS, physical component scale

Characteristic	Total (N=57)	Improved (N=40) | 70%	Recurrence (N=17) | 30%	p-value
Age at first MRI (years)				
Median (interquartile range)	31 (25-45)	31 (25-44)	31 (26-47)	0.85
Range	15-56	15-54	16-56	
Gender (%)				
Female	51 (89%)	36 (90%)	15 (88%)	0.85
Male	6 (11%)	4 (10%)	2 (12%)	
Preoperative SF-12 PCS				
Mean (interquartile range)	30.8 (24.0-35.2)	28.7 (22.4-34.3)	35.6 (30.3-36.4)	0.013
Range	11.6-53.4	11.6-53.4	17.0-55.5	
MRI cine score (%)				
Cine A	15 (26%)	10 (25%)	5 (29%)	0.73
Cine B	31 (54%)	23 (58%)	8 (47%)	0.48
Cine C	8 (14%)	6 (15%)	2 (12%)	0.75
Cine D	1 (2%)	0 (0%)	1 (6%)	0.13
Cerebellar ectopia (mm)				
Mean (interquartile range)	10.1 (7.0-12.0)	11.0 (7.8-14.2)	8.2 (6.0-10.8)	0.061
Range	0-23.0	0-23.0	0-15.5	
Syringomyelia present (%)				
Present	16 (28%)	11 (28.5%)	5 (29%)	0.89
Absent	41 (72%)	29 (72.5%)	12 (71%)	
Symptoms (%)				
Headache	50 (88%)	36 (90%)	14 (82%)	0.43
Limb pain	12 (21%)	11 (28%)	1 (6%)	0.069
Numbness	7 (12%)	5 (13%)	2 (12%)	0.94
Limb paraesthesia	20 (35%)	14 (35%)	6 (35%)	0.98
Limb weakness	8 (14%)	6 (15%)	2 (12%)	0.75
Vertigo	13 (23%)	8 (20%)	5 (29%)	0.45
Nystagmus	2 (4%)	2 (5%)	0 (0%)	0.36

As seen in Table 3, the five-fold cross-validation model performance using a deep learning CLAM model trained and validated on MRI data including augmentation achieved a mean sensitivity of 72.5% (SD: 16.3), specificity of 48.3% (SD: 19.0), accuracy of 64.7% (SD: 7.3), F1 score of 0.74 (SD: 0.07), and an area under the receiver operating characteristic (ROC) AUC curve of 0.68 (SD: 0.19).

**Table 3:** Five-fold cross-validation performance metrics of different models: imaging only (MRI), clinical features only in isolation and combination, and combined imaging and specific clinical features All values are means and SDs calculated on the five runs. All models incorporating imaging analysis included data augmentation for training and validation, while the test dataset was not augmented. MRI, magnetic resonance imaging; PCS, physical component scale; ROC, receiver operating characteristic; AUC, area under the curve; SD, standard deviation

	Sensitivity (SD)	Specificity (SD)	Accuracy (SD)	F1 score (SD)	ROC AUC (SD)
MRI	72.5% (16.3)	48.3% (19.0)	64.7% (7.3)	0.74 (0.07)	0.68 (0.19)
All clinical features	65.0% (20.5)	60.0% (19.9)	63.5% (13.3)	0.70 (0.15)	0.61 (0.16)
PCS	60.0% (13.7)	63.3% (24.7)	61.1% (11.4)	0.68 (0.11)	0.69 (0.22)
Symptoms	47.5% (18.5)	23.3% (13.7)	40.6% (11.0)	0.52 (0.15)	0.38 (0.16)
Cerebellar ectopia	52.5% (16.3)	53.3% (22.5)	52.9% (10.3)	0.60 (0.12)	0.66 (0.18)
Age and gender	45.0% (14.3)	28.3% (18.3)	40.2% (8.8)	0.50 (0.11)	0.34 (0.08)
CINE	55.0% (20.9)	30.0% (24)	47.3% (18.2)	0.58 (0.17)	0.40 (0.19)
PCS + cerebellar ectopia	67.5% (16.8)	65.0% (26.6)	66.8% (11.0)	0.73 (0.10)	0.71 (0.22)
MRI + PCS + cerebellar ectopia	67.5% (11.2)	63.3% (24.7)	66.5% (12.2)	0.74 (0.10)	0.71 (0.19)

Of the models developed using clinical features, only models using cerebellar ectopia and PCS resulted in isolation and achieved an AUC greater than 0.65. Across the five-fold cross-validation, the model trained using only PCS result achieved a mean sensitivity of 60.0% (SD: 13.7), specificity of 63.3% (SD: 24.7), accuracy of 61.1% (SD: 11.4), F1 score of 0.68 (SD: 0.11), and an AUC of 0.69 (SD: 0.22). The model trained using only cerebellar ectopia achieved a mean sensitivity of 52.5% (SD: 16.3), specificity of 53.3% (SD: 22.5), accuracy of 52.9% (SD: 10.3), F1 score of 0.60 (SD: 0.12), and an AUC of 0.66 (SD: 0.18). A combined model with PCS and cerebellar ectopia features was trained and achieved a mean sensitivity of 67.5% (SD: 16.8), specificity of 65.0% (SD: 26.6), accuracy of 66.8% (SD: 11.0), F1 score of 0.73 (SD: 0.10), and AUC of 0.71 (SD: 0.22). Models trained using age and gender alone, cine MRI score alone, and all clinical features combined had mean AUC performances between 0.34 and 0.61.

Finally, a combined clinical and imaging model was trained using MRI, preoperative PCS results, and the extent of cerebellar ectopia. This model achieved a sensitivity of 67.5% (SD: 11.2), specificity of 63.3% (SD: 24.7), accuracy of 66.5% (SD: 12.2), F1 score of 0.74 (SD: 0.10), and an AUC of 0.71 (SD: 0.19). This performance was similar to the imaging-only model that had an AUC of 0.68 (SD: 0.19). 

## Discussion

To our knowledge, this is the first study to utilize machine learning and deep learning in the prediction of recurrence of symptoms postoperatively for CM1 patients undergoing surgical management. Determining at-risk patients is critical as up to a third of patients experience a recurrence of their CM1, with 4-13% of patients requiring reoperation [1-3]. The model that combined volumetric MRI data with clinical features including the preoperative PCS result and extent of cerebellar ectopia performed best and had a mean F1 score of 0.74 (SD: 0.10) and a mean ROC AUC of 0.71 (SD: 0.19) on testing. Currently, the CM1 definition is based on tonsillar herniation in isolation and there are multiple studies utilizing AI to build on this definition and either improve the identification of tonsillar herniation or determine new clinically relevant neuroimaging and clinical features [7,13-15]. However, the prediction of the recurrence of symptoms aims to aid in better patient selection to prevent the recurrence of symptoms and potential reoperation. Our recurrence rate of 30% (17/57) is consistent with the literature, and the high proportion of female participants is not unexpected given the well-established increased prevalence of adult CM1 in females [16].

In terms of the prediction of symptom recurrence, several studies have explored radiological predictors with inconsistent results. Wang et al. showed that a smaller clivus-supraocciput angle of less than 96º is a predictor of clinical or radiographic outcomes following surgical decompression [17]. McGirt et al. found a correlation between hindbrain CSF flow and outcome after surgical decompression of CM1 where the presence of decreased CSF flow both ventral and dorsal to the cervicomedullary junction was associated with improved response to hindbrain decompression for CM1 in children [12]. They also demonstrated that a cine phase-contrast MRI may be a useful tool for surgical risk stratification and identifying patients that may be optimal surgical candidates and that combined ventral and dorsal hindbrain CSF flow pathology may better predict response to posterior fossa decompression compared to dorsal CSF flow pathology alone. In their study, while symptom recurrence did not differ as a function of the degree of tonsillar ectopia, patients with both ventral and dorsal decrease in CSF flow after surgery had reduced recurrence rates and patients with just dorsal decrease flow had larger recurrence rates. Liu et al. found that posterior cranial fossa morphology did not predict response to posterior fossa decompression in CM1 [18]. Some studies have found that syrinx size change and tonsillar tip distance from foramen magnum do not have a predictive association with outcome or recurrence, while others have found an association between syrinx size reduction and postoperative symptomatic improvement [19]. Studies looking at clinical features that predict CM1 recurrence have found that longer duration of headache, frontal headache, and vertigo independently increased the likelihood of symptom recurrence after decompression, and preoperative upper limb numbness and weakness are unfavorable factors predicting the outcome of posterior fossa decompressions in patients with CM1 [12,18]. Prognostic tools including the Chiari Severity Index and a points-based algorithm of Thakar et al. have been developed to consolidate clinical and neuroimaging features that predict recurrence; however, Feghali et al.’s external validation study concluded that these scoring systems failed to provide prediction value of clinically meaningful improvement following decompression [4-6].

The optimal model trained in this study used the CLAM machine learning framework, utilized the whole volumetric MRI data, and combined it with clinical data. It provides a machine learning methodology for further studies to explore with the deep learning technique approach removing the need for manual feature extraction. CLAM is a multiple-instance deep learning-based weakly supervised methodology that allowed for the training of a whole-image classifier using MRI volumes that only had labels for the whole volume, rather than by diagnostic slice or subregion [9]. While some studies have reported radiological predictors of surgical outcome, other studies have shown that no specific parameters on preoperative MRI evaluation were predictive of the outcome of surgery, emphasizing the importance of applying clinical information in surgical decision-making [19]. In our study, we used worsening in the postoperative SF-12 PCS to capture recurrence. Godil et al. found that of the patient-reported outcome instruments (VAS Head, VAS Neck, NDI, and HDI for pain and disability; the SF-12 PCS, SF-12 MCS, Zung, and EQ-5D), the SF-12 PCS (together with the EQ-5D) was the most valid and responsive measure to postoperative improvement in pain, disability, and quality of life in patients with CM1 [20].

Despite a 10-year study period and a relatively high-volume institution for CM1 surgical management, the main limitation of this study was the size of the dataset of only 57 patients. The random splitting of data for the cross-validation and testing also represented a potential source of random error but the five-fold cross-validation aimed to mitigate this. Data augmentation accounting for class imbalance also helped to minimize the impact of the small dataset. Further data collation could allow for unsupervised learning with saliency mapping to be applied to better understand the neuroimaging features that contribute to poor postoperative outcomes.

## Conclusions

The results of this study represent the first step toward the development of a machine learning model that utilizes preoperative neuroimaging and clinical data to aid surgical outcome prognostication for CM1 patients and, therefore, improve patient selection for surgery. Further studies utilizing a similar deep learning approach with a larger sample size are needed to improve the performance of the predictive model.
